# Barriers and facilitators to accessing sexual and reproductive health services for people with severe mental illness: a systematic review

**DOI:** 10.1007/s00127-025-02844-0

**Published:** 2025-02-28

**Authors:** Matilda Brown, Emma Tassie, Sophie Carlisle, Elana Covshoff, Amy Ronaldson, Julie Williams, Shubulade Smith, Kylee Trevillion, Elizabeth Hughes, Margaret Heslin

**Affiliations:** 1 Health Service and Population Research Department, Institute of Psychiatry, Psychology and Neuroscience, King’s College London, London, UK; 2 Guy’s and St, Thomas NHS Foundation Trust, London, UK; 3 Centre for Implementation Science, King’s College London, London, UK; 4 Institute of Psychiatry, Psychology and Neuroscience at King’s College London, London, UK; 5 Department of Nursing and Public Health, School of Health and Life Sciences, Glasgow Caledonian University, Glasgow, UK

**Keywords:** Sexual health, Severe mental illness, Barriers, Facilitators, Access

## Abstract

**Purpose:**

Despite increased prevalence of sexual and reproductive health problems among people with severe mental illness (SMI), uptake of sexual and reproductive healthcare in this group is poor. The reasons for this are unclear. Therefore, this review aimed to identify the barriers and facilitators to accessing sexual and reproductive health services from a service user perspective.

**Methods:**

Three electronic databases were searched using key words for “sexual health” and “SMI”. Data were screened and extracted by two independent reviewers. The Joanna Briggs Institute Critical Appraisal Tools were used to assess quality of included studies.

**Results:**

Five studies were included and underwent a narrative synthesis. They were on access to HIV care (n2), access to family planning methods (n2) and access to general sexual healthcare (n1). Barriers relating to HIV care included cost; barriers relating to family planning included lack of awareness and not considering the issue; barriers to general sexual healthcare included psychotic symptoms, mental health prioritisation, stigma, lack of sexual health focus in mental health programs, difficulty initiating conversations, knowledge, culture/religion/ethnicity, and finances.

**Conclusions:**

Studies which examined access to HIV and family planning services did so in a way that limited participant responses. While only one study examined barriers and facilitators to accessing generic sexual health services, it did so robustly, although it focussed solely on young women and provided limited data on facilitators. Future work should focus on examining barriers, and facilitators, to accessing sexual healthcare in all people with SMI to better identify and address these challenges.

*PROSPERO ID* CRD42023414740.

**Supplementary Information:**

The online version contains supplementary material available at 10.1007/s00127-025-02844-0.

## Introduction

People with severe mental illness (SMI, defined here as schizophrenia and related disorders) have higher morbidity and mortality compared to the general population [1, 2]. This contributes to people with SMI having a reduced life expectancy compared to the general population of up to almost 18 years in some groups [3]. For this reason, increasing attention has been given to the physical health of people with SMI. However, although it is an important aspect of physical health, the sexual health needs of people with SMI are often neglected [4, 5].

Sexual health is defined here as a state of physical, emotional, mental and social wellbeing in relation to sexuality [6] and includes reproductive health. People with SMI are at a higher risk of having poor sexual and reproductive health or unmet needs, and are at higher risk of blood borne viruses [7, 8]. A systematic review and meta-analysis of HIV, hepatitis B and hepatitis C rates reported that these blood-borne infections were more prevalent in people with SMI compared to the general population [4]. In fact, a recent study [9] found that HIV prevalence was approximately double that of the general population at 20.3 per 1,000 in people with schizophrenia. Furthermore, people with SMI have an increased risk of having other sexually transmitted infections (e.g., syphilis [10]). In terms of reproductive health, people with SMI are more likely to experience difficulties including unintended pregnancies [11], recurrent miscarriage, abortion, gynaecological diseases, and reproductive cancers [12], and are more likely to experience sexual assault [13] and sexual dysfunction [14].

Due to the higher risk of sexual and reproductive ill health and unmet needs in people with SMI, it is vitally important that this population receive sexual health care in a timely fashion. However, Hughes et al. [15] reported that only 54% of people with SMI had ever accessed sexual health services. Walkup et al. [16] has also reported that the uptake of sexual health care is lower in people with SMI and care is often delayed. This can prolong illness, lead to more severe disease, reduce quality of life, and increase health care costs. However, the reasons for the lack of uptake or delays in sexual healthcare among people with SMI is unknown. Therefore, we cannot yet determine the best course of action to improve access to sexual and reproductive health care for people with SMI.

De Hert et al. [17] examined the barriers to the recognition and management of physical diseases in service users with SMI and noted different categories of barriers. These included barriers related to the service-user, illness, treatment, psychiatrist, other physicians, and services in general. Although it is useful to examine all types of barriers to care, service user- and illness-related barriers are particularly important to focus on as these types of barriers may not be obvious to healthcare professionals. Therefore, examining barriers and facilitators to accessing care from a service user perspective is incredibly important.

This study aimed to conduct a systematic review on the barriers and facilitators to accessing sexual and reproductive health care for people with SMI, from a service user perspective.

## Materials and methods

This systematic review followed the Preferred Reporting Items for Systematic Reviews and Meta-Analysis (PRISMA [18]; see online appendix for PRISMA checklist). Prior to formal screening, this review was registered on PROSPERO under “*Barriers and Facilitators to Accessing Sexual Health Services for People with Serious Mental Illness: A Systematic Review”* (ID: CRD42023414740, 18/04/2023).

### Search strategy

Accessed via OVID, three electronic databases were searched on 10/01/2025 from inception to the date of the searches: APA PsychInfo, Medline, and EMBASE, selected due to their large literature bases of medical and psychological research. To maximise the scope of the search, no limitations on year of publication, study design, or research type were imposed. Forward and backward citation tracking was also used. Due to resource limitations, grey literature was not searched.

#### Search terms

Two blocks of search terms were developed (see online appendix for full details). The first included terms relating to SMI, e.g. ‘severe mental*’, ‘psychosis*’, and ‘schizophreni*’. Terms relating to mental health professionals were also included in this block e.g., ‘mental health nurse*’, as mixed samples of professionals and clients were eligible for inclusion if service user perspectives could be derived from results.

The second block referred to sexual and reproductive health services. Only generic sexual and reproductive health service related terms were included as the focus of this review was generic services. Specialist services such as sexual assault or sexual dysfunction services were not included as it was outside the scope of this review. Terms such as ‘genitourinary medicine*’, ‘sexual health service*’ and ‘contracept*’ were included. The term ‘sexual health*’ was also included. Within-block terms were connected by the Boolean operated ‘OR’, and between-block terms with ‘AND’. Subject Headings were also included (see online appendix for full details).

### Selection criteria

Studies were selected for inclusion if:

- The sample included people with SMI, excluding mixed diagnostic groups, but including samples with health professionals if presented with separate service user data.
- AND the paper included data/information from a service user perspective on why they are not accessing sexual health services (e.g., attending sexual health clinics) or receiving sexual health care (e.g., getting contraceptives).
- AND the paper was published in English.

#### Sample

Aligned with previous research [4], SMI was defined as disorders affected by psychosis: bipolar disorder, schizophrenia, schizoaffective disorder, and other psychosis-related illnesses. Diagnoses needed to be reported using an established diagnostic manual or validated diagnostic criteria, such as the ICD, DSM, or RDC [19–21]. If diagnostic systems were unnamed, but diagnoses were derived from medical records, these articles were included under the assumption that formal diagnoses had been given. There were no age limits on samples of people with SMI to remain over-inclusive, as sexual health services are accessible to all ages in the UK. Mixed samples, including common and severe mental health problems, and samples in which service user and healthcare professional data were merged were excluded due to specific focus on service user perspectives. Reviews and books were excluded.

#### Phenomenon of interest

As mentioned above, the focus of the review was generic sexual and reproductive health services. The wording of the selection criteria allowed papers to be included for review if they referred either to general service access or access to specific interventions. The term “data/information” ensured papers with a broad focus on SMI and sexual health services were reached in screening. For example, those qualitatively investigating experiences of sexual health services for people with SMI may contain information on access within their findings.

### Screening procedure

Complete references from OVID were exported and de-duplicated in EndNote before being transferred to Rayyan (http://rayyan.qcri.org). One researcher (MB) completed the title and abstract screening, a second reviewer (ET/MH) double screened a random 10%. Full text articles were double screened by two reviewers independently (MB & ET/MH). The agreement rate for title/abstract screening was 99%. The agreement rate for full text screening was 99%. Disagreements were discussed and resolved.

### Data extraction

Data was extracted by two reviewers independently (ET/MH & MB), and disagreements were discussed and resolved. Data extracted included: authors, year of publication, title, research location and setting, study design, SMI sample size and participant characteristics, aspect of sexual health, measurement of outcome of interest, and findings on access to sexual health services. The relevant data was input into an Excel document before tabulating. As access to sexual health care was not the sole or primary outcome in any of the articles included for review, only information relevant to our research questions was extracted.

### Critical appraisal

As the study design was unspecified in the selection criteria, three versions of the Joanna Briggs Institute (JBI) Critical Appraisal Tools [22] were used: analytical cross-sectional studies, case control studies, and qualitative studies. Often used in systematic reviews, these checklists follow the same system of marking, “yes”, “no”, “unclear”, “not applicable” to assess the validity and reliability of an article across various domains [23]. As performed in previous work [24], a score of 1 was provided for each “yes” mark. There were ten items for case–control studies and qualitative studies, and eight for analytical cross-sectional studies, producing maximum scores of ten, ten and eight, respectively. A score of 70% or higher was deemed high-quality evidence. On items referring to measurements of outcome, the quality appraisal was performed in the context of this review’s outcome of interest, i.e., reasons why people with SMI are not accessing sexual health care. Two reviewers (MB & ET/MH) independently performed the critical appraisal and disagreements were discussed and resolved.

### Data analysis

Study selection and study characteristics are presented. Given the small number of eligible studies and heterogeneity of methods, a narrative synthesis was conducted. Studies are described and discussed in the context of quality and limitations.

## Results

### Study selection

**Fig. 1:**
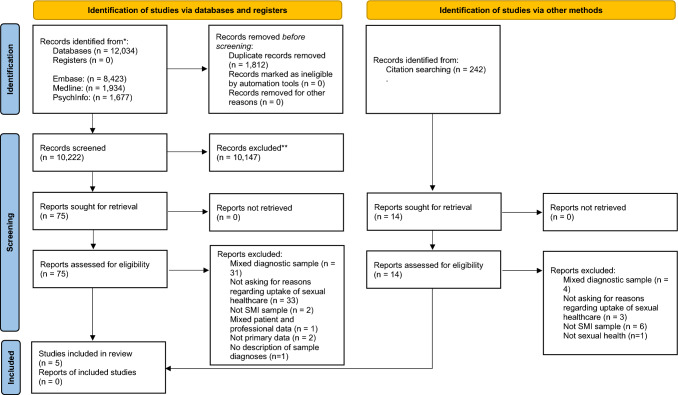
PRISMA 2020 flow diagram

The total number of papers identified was 12,034. After removing duplicates, 10,222 unique references were eligible for title and abstract screening. Of these, 10,147 were excluded, leaving 75 to be examined by full text. Five studies were deemed appropriate for inclusion in this review [25–29]. Reasons for excluding 70 full texts are presented in the PRISMA flowchart in Fig. 1.

### Study characteristics

The key characteristics and quality appraisal scores of the included studies are reported in Table 1. In one case [26], the geographical location of the research was not stated, thus, it was assumed based on the author’s backgrounds. There was no other missing information across the papers. Publication dates ranged between 1998 and 2024. Three were from studies based in the US, one in Canada, and one in South India. Three studies had female-only samples, whilst the other two were majority male. Two focussed on HIV care, two on family planning, and one focussed on general sexual and reproductive healthcare. One study solely focussed on schizophrenia and one included people with psychosis more generally, whilst the others also included diagnoses of bipolar disorder, schizoaffective disorder, and major depression with psychotic features.

**Table 1:** Key characteristics of the five studies included for review

Authors Year Title	Research Location Setting	Study Design	SMI Sample Size and Participant Characteristics	Aspect of Sexual Health	Measurement of Outcome of Interest	Findings on Access to Sexual Health Services	JBI Quality Appraisal Checklist and Score
*Papers Investigating Access to HIV Care*							
Fremont et al 2007 Differences in HIV care between patients with and without severe mental illness	Los Angeles and New York City, USA Mental Health Clinics and Programs for people with comorbid SMI and HIV	Case–control, structured interview Compared regional outcomes for people with SMI and HIV to people with HIV without SMI	SMI participant total n = 294 Age: majority (64%) 35–49 years old Gender Identification: majority male (70%) Diagnoses: schizophrenia, schizoaffective disorder, bipolar disorder, major depressive disorder with psychotic features, proportions of diagnoses not reported. Identified by records from mental health agencies Ethnicity: White 25%, Black 51%, Hispanic 18% and Other 6%	The impact of SMI on HIV care Examined the barriers to obtaining HIV care	SMI participant responses to 2 single items on barriers to HIV care, developed from the HCSUS survey: “*During the last six months, did* *you ever need physical health care but could not get it?”* and “*In the past six months, have* *you ever had to go without health* *care that you needed because you* *needed the money for food, clothing,* *housing, *etc*.?”*	People with SMI were significantly more likely than people without SMI to report not being able to obtain HIV care In Los Angeles, 18% of people with SMI and HIV reported difficulty obtaining care, compared to 5% of people without SMI (p-value < 0.001). In New York, 16% of people with SMI and HIV reported being unable to obtain care, compared to 7% of people without SMI (p-value < 0.02)	Case–control checklist, score 8/10 (80%)
Sullivan et al 2006 Co-location of Health Care for Adults with Serious Mental Illness and HIV	Los Angeles, USA Mental health agencies and programs	Secondary analysis of data (Bogart et al., 2006#), structured interview only using data from SMI participants	Total n = 118 Age: majority (62%) 35–49 years old Gender Identification: 82% male Diagnoses: schizophrenia or schizoaffective disorder (40%), bipolar disorder (36%), psychotic depression (24%), reported from mental health agencies Ethnicity: 41% white, 40% African American, 14% Hispanic, 5% other	HIV care Comparing outcomes for people with SMI who receive mental health and HIV care at the same or different sites	Three-item index of barriers to HIV care from the HCSUS survey: *“the proportion of subjects who, in the last 6 months believed they could not get needed* *medical care, went without care because of lack of money, or went without food because they needed money for care”.* Responses were totalled and calculated to fit a barriers scale used in Bogart et al. (2006)	Less than 1 in 5 participants with SMI reported any barriers to care (17%). No significant differences on this measure between single or different sites of SMI and HIV care	Analytical cross-sectional checklist, 5/8 (62.5%)
*Papers investigating access to family planning*							
Miller & Finnerty 1998 Family planning knowledge, attitudes and practices in women with schizophrenic spectrum disorders	Did not state, assumed USA-based Inpatient and outpatient services in a teaching hospital	Case–control, semi-structured interview	Total n = 94 SMI n = 44 Age: range 18–45, mean 30.8 years Gender Identification: 100% female Diagnoses: 26% schizophrenia, 28% schizoaffective bipolar, 46% schizoaffective depression. Identified using Schizophrenia-Lifetime Version (SADS-L) and Research Diagnostic Criteria Ethnicity: Missing for the SMI group only	Family planning—collected information about reproductive health history, birth control knowledge, practices, and attitudes	Reasons why participants did not use birth control (in year prior to interview) though not desiring pregnancy. Asked in the Family Planning Interview schedule, questions modified from instruments used in other texts	*Didn’t expect to have sex: 46.5% *Didn’t think about birth control or pregnancy while having sex: 45.2% *Side-effects from birth control: 41.9% *Didn’t think I could get pregnant: 35.7% *Sort of wished for pregnancy: 35.7% *Hard to remember birth control: 35.7% *Didn’t get around to it: 31.0% *Partner doesn’t like birth control: 28.6% *Too hard to get birth control: 25.0% *Hate pelvic exams: 23.8% *Someone might find birth control: 21.4% *Inconvenient to use: 19.0% *Birth control doesn’t work: 15.0% *Not romantic: 13.6% *Against my religious beliefs: 4.5% *Never heard of birth control: 2.4%	Case–control checklist, 6/10 (60%)
Sethuraman et al 2019 Knowledge, Attitude, and Practice Regarding Contraception among Women with Schizophrenia: An Observational Study from South India	South India, does not state exact locality Outpatient clinic of the Department of Psychiatry	Observational, questionnaire, delivered by semi-structured interview	Total n = 96 Age: mean age 33.5 Gender Identification: 100% female Diagnoses: all had an ICD-10 diagnosis of schizophrenia Ethnicity: not reported, all South Indian residents	Family Planning – knowledge, attitudes, practice of contraception and family planning methods	Reasons for not using contraception and/or delaying pregnancy. Questionnaire based on the NFHS-3, referenced in text	Approximately 1 in 3 of participants were not using contraception 15% had unmet contraceptive needs Reasons for not using contraception included: *Lack of adequate awareness: 35.5% *Fear of side effects: 25.8% *Wants to have a male child/another child: 45.2% *Did not receive information about family planning in mental health services: 32.3% *Social reasons: 9.6% *Not expecting to have sex: 9.6% *Difficulty in planning ahead about contraception: 3.2% *Difficulty in accessing family planning services: 3.2% *Felt her view was not considered important by her family: 3.2% *Inconvenient to use: 3.2%	Analytical cross-sectional checklist, 5/8 (62.5%)
*Papers investigating general sexual healthcare access*							
Barker et al. 2024 Experiences of Sexual and Reproductive Health Care Access for Women and Nonbinary People With Early Psychosis: Towards an Integrated Perspective of Service Users and Clinicians	Toronto, Ontario, Canada Women’s College Hospital, an outpatient academic hospital with specialized SRH programs, partnered with the Centre for Addiction and Mental Health, an academic psychiatric hospital, and the Canadian Mental Health Association -Toronto, a community mental health organization	A qualitative study using semi-structured qualitative interviews	Total: 19 service users Age: 18–31 years Gender: Predominantly cisgender women (cisgender women n = 15, transgender women n = 2, nonbinary/gender-diverse n = 2) Diagnoses: People with psychotic disorders Ethnicity: White n = 6 (31.6%), Black, Asian, Indigenous, Hispanic, or mixed race/ethnicity n = 13 (68.4%)	Experiences of sexual and reproductive healthcare access and utilization	Themes from the thematic analysis of semi-structured interviews	Barriers include: * Psychotic symptoms * Mental health Prioritisation * Mental health stigma * Lack of sexual health focus in mental health programs * Difficulty initiating conversations * Knowledge gaps * Cultural, religious and ethnic barriers * Financial barriers Facilitators include: *Siloed services	Qualitative checklist, 10/10 (100%)

^#^Bogart et al. (2006) cited in main reference list at the end of this report

### Methodological quality

The quality assessment (Table 1) indicated that two studies had a high quality [25, 29], and three had a medium to low quality [26–28]. The studies were mostly limited in their measures of barriers and facilitators to accessing sexual health care, as well as their identification and handling of confounding variables.

### Barriers to accessing sexual healthcare

The results have been grouped by the type of sexual health service each study assessed: access to HIV services; access to family planning methods; and access to general sexual healthcare.

#### Access to HIV care

Two studies investigated access to HIV care [25, 28], both US-based and included a range of diagnoses under SMI. Diagnoses were gathered from medical records and agencies. Their samples were majority male and ranged from 18 to 50 years old.

Fremont et al. [25] adopted a structured interview approach to examine experiences of HIV care for people with SMI versus those without. Their main study outcomes compared HIV care between groups based on residency in Los Angeles or New York City, considering barriers to HIV care as a secondary outcome. The two questions asked about barriers to care regarded needing physical healthcare but being unable to “get it” and going without health care due to cost of living issues (See Table 1). They reported that people with SMI are less likely to obtain and more likely to decline HIV care due to cost compared to people with HIV and no SMI but did not report exact figures for each group.

Considering risk of bias assessment, Fremont et al. [25] scored 8/10 (80%), mostly due to sound matching of cases and controls, measurements of exposure, and handling of confounding variables. However, their assessment of barriers to HIV care remains questionable. The authors measured barriers to HIV care with two single items from the national HIV Cost and Services Utilisation Study (HCSUS) [25]. These were pre-defined to ask about cost, one item not being mentioned until their discussion. Adaptations of these items were not evidenced as validated, and the authors failed to mention any reliability check of participant responses, therefore hindering the internal validity and inter-rater soundness of their findings.

Sullivan et al. [28] undertook a cross-sectional study concerning the difference in utilisation and satisfaction of healthcare when receiving HIV and mental health support at single or separate sites, comparing SMI to non-SMI groups. They assessed hospital experiences, communication between separate sites, and barriers to obtaining care. Like Fremont et al. [25], their assessment of barriers to HIV care was taken from the HCSUS survey, using a three-item index of closed questions concerning the proportion of participants who believed they could not obtain care due to insufficient funds and other priorities. They found that some participants with SMI reported experiencing cost as a barrier to HIV care (17%). On the one hand, these findings can be perceived as positive outcomes, yet their sample involved clients who had already gained access to services, and these findings must be considered in the context of the risk of bias.

A score of 5/8 (62.5%) was given to Sullivan et al. [28], representing mid-quality evidence. Their handling of confounding variables and method of measuring communication between HIV and mental healthcare sites was unclear. Additionally, although their closed questions on barriers to HIV care indicated yes/no responses, the proportion of agreement on each item, for example, not accessing care due to lack of money versus needing food, are omitted. Thus, findings remain somewhat limited.

#### Access to family planning methods

The oldest and most recent studies focussed on family planning practices for women with SMI [26, 27], both adopting semi-structured interview designs. Whereas Miller and Finnerty [26] included a range of diagnoses assessed by the RDC [21], Sethuraman et al. [27] only included women with an ICD-10 [19] diagnosis of schizophrenia. Both reported similar age ranges of participants (See Table 1).

Miller and Finnerty [26] compared women with and without SMI. They gathered data on sexuality and reproductive experiences, including attitudes and practices of birth control methods. Their interview schedule took instruments from previous work, creating a “Family Planning Interview”. After receiving spontaneous responses from women with SMI about why they were not using contraception, the researchers provided participants with 16 pre-defined reasons, asking women to indicate which applied to them. Reasons that were statistically more common in women with SMI than those without were “Didn’t get around to it” at 31% versus 12%, and “Too hard to get birth control” at 25% versus 0%. In relation to birth control being hard to get, the authors do not report any exploration of this item. “Birth control doesn’t work” was also higher at 15% versus 2% but not statistically different between the groups. The most common reason among those with SMI were “Didn’t expect to have sex” (46.5%) and not “Didn’t think about birth control or pregnancy while having sex” (45.2%). All reasons reported can be found in Table 1.

In terms of quality assessment, the interviewers in Miller and Finnerty’s [26] study were trained by professionals, and the responses of women with SMI were checked with clinicians, boosting the reliability of their findings. However, Miller and Finnerty [26] failed to cite where the 16 coded answers were derived from, and did not report the details of the qualitative accounts from their participants. Therefore, potentially core information on barriers to contraception use was omitted. Consequently, some risk of bias is introduced when deeming these results from the perspectives of women with SMI. Their study scored 6/10 (60%) on the risk of bias assessment, representing mid-quality evidence.

Whilst the previous papers were all US-based, Sethuraman et al. [27] examined birth control attitudes and practices among women with SMI in South India. Their interview protocol was adapted from the National Family Health Survey (NFHS-3 [30]), however, Sethuraman et al. [27] do not explicitly state whether the question on reasons for not using contraception elicited open-ended or closed responses. Around a third of participants were not using contraception at the time of data collection, and almost 15% had unmet contraceptive needs. Wanting to become pregnant (45.2%), lack of awareness (35.5%), and not receiving information about family planning from mental health services (32.3%) were the most frequently reported reasons for not using contraception.

Sethuraman et al. [27] scored 4/8 (50%) on the risk of bias assessment, indicating relatively poor quality of evidence. Whilst they failed to indicate whether they collected spontaneous responses from participants, semi-structured interviews are an exploratory tool usually involving qualitative data gathering [31]. As such, open-ended answers likely to be rich in validity appear to be missing in this report. Furthermore, one author collected and presumably interpreted their data, yet the process and reliability of evidence synthesis remains ambiguous.

#### Access to general sexual healthcare

One study by Barker et al. [29] investigated access to general sexual healthcare. This was with women (cisgender, transgender and nonbinary/gender-diverse women) with psychosis in Canada. It included information from both service users and clinicians, although the latter is not discussed here*.* This was a qualitative study based on semi-structured interviews. Although the focus was experiences of sexual and reproductive healthcare access and utilisation, this naturally raised issues around barriers and facilitators to care.

Several barriers to care were highlighted. Psychotic symptoms, particularly paranoia and disorganisation, hindered service users’ ability to trust professionals, arrange appointments, and engage in care offered. While not explicitly described as a barrier, some service users reported prioritizing mental health care above other health needs, which can lead to neglect of sexual health concerns.

Mental health stigma was raised as an issue by participants, especially in relation to decisions to have children, and the discomfort around discussing sex. Service users described a lack of attention to sexual and reproductive health in mental health programmes and struggled to initiate conversations about sexual health. When issues were raised, participants reported solutions were not always provided by their care providers.

A lack of sexual health knowledge was also noted. Some respondents turned to physicians for advice, while others reported using the internet and media for information. Culture, religion, and ethnicity were raised by multiple service users as barriers to access care. Financial barriers, particularly in relation to contraception, were also raised.

Finally, service users described challenges in accessing specialized services for sexual difficulties like dyspareunia, but the paper did not provide detailed descriptions of these challenges. The study received a risk of bias score of 10/10.

### Facilitators to accessing sexual health services

Only Barker et al. [29] reported on facilitators to accessing sexual health services for people with SMI. As discussed above, barriers and facilitators were not the focus of this paper, however, in the process of exploring experiences of sexual and reproductive healthcare, some facilitators were highlighted. Service users discussed how siloed mental health and sexual health services allowed a degree of anonymity that made it easier for them to access care. This was the only facilitator discussed.

## Discussion

### Key findings

This review identified two studies which included data on barriers to accessing HIV care, two studies which included data on barriers to accessing reproductive care, and one which included data on experiences of sexual and reproductive healthcare in general—all from a service user perspective. The studies on HIV access both highlighted cost as a barrier. The reproductive care studies highlighted a number of barriers, including not thinking about it, lack of awareness, difficulty getting access to contraceptives and a lack of belief that contraceptives work. The study on general sexual and reproductive healthcare highlighted a list of barriers including psychotic symptoms, mental health prioritisation, stigma, lack of sexual health focus in mental health programs, difficulty initiating conversations, knowledge, culture/religion/ethnicity, and finances. Only one study included facilitators of accessing sexual and reproductive healthcare and that only mentioned one facilitator which was siloed mental health and sexual health services.

Both studies on access to HIV care used closed questions on barriers and focussed on cost only [25, 28]. This pre-specification leaves no room for exploring other obstacles people with SMI might experience when trying to access HIV services. Similarly, Miller & Finnerty’s [26] exploration of barriers to accessing reproductive care used a pre-defined list without describing where this came from, and Sethuraman et al. [27] was unclear whether they used open or closed questions. Again, this pre-specification leaves no room to further explore the issues from a service user perspective.

Further, four of the five studies examined access to only two specific aspects of sexual and reproductive healthcare, leaving access to broader areas like the use of sexual health clinics unexplored. Only one study [29] explored sexual and reproductive healthcare in general. Barker et al. [29] explored experiences of sexual and reproductive healthcare which included barriers to care. They did so in qualitative semi-structured interviews with service users, allowing service users to explore the topic from their own perspective without being constrained by closed-ended questions. Possibly due to this methodological approach, the researchers were able to identify a greater number of barriers than those found in the other four studies. Additionally, the methods of this study were robust, reflected by the high risk of bias score. However, the study included only women aged 18–31. It is likely that men and older age groups face a different set of barriers which have not been explored here.

Other factors important to consider include the location of the studies. Four out of the five studies come from the US and South India, which have predominantly private health care systems, making it difficult to generalise to countries with predominantly public health services or mixed health services. Additionally, only one of the included studies regarded access to sexual health services as the primary focus of their research, asking these questions as secondary to, or alongside, other points of interest. Finally, the data quality was mostly low or moderate, reducing our confidence in the findings.

## Strengths and limitations of review

This review had several strengths. The databases used contain a breadth of published research, the over-inclusive search strategy allowed a large number of studies to be reached, and the use of a second reviewer at all screening stages boosted the reliability and reproducibility of results. Moreover, this is the first review exploring the important topic of access to sexual health services for people with SMI.

However, findings should also be considered in the context of multiple limitations. This review focussed on generic sexual and reproductive services and did not include search terms related to specialist sexual health provision such as sexual violence and sexual dysfunction services. These were outside the scope of this review but access to these services is also of vital importance for people with SMI and future research should include this.

A further limitation of this review could be the characterisation of SMI as disorders affected by psychosis. This definition was chosen due to its use in successful reviews of the same genre [4]. However, potentially relevant papers were excluded. For example, Lawley et al. [32] had an expansive definition of SMI when qualitatively investigating barriers to reproductive care that included people with psychosis but also people with depression and post-traumatic stress disorder. Their findings align with those produced in this review: finances, knowledge of clinics, and difficulty locating sexual health services all influence access. Nonetheless, they also provided deeper, nuanced accounts, such as women with SMI reporting prescribed contraception as a non-priority consideration and difficult to comply with [32]. These reasons present an interesting interaction between the nature of SMI and utilisation of sexual health care missed in this review. Although it stands that service user perspectives seem muted across the literature looking into access to sexual health services for people with SMI, perhaps our definition of what constitutes severe mental illness also limited our ability to locate such research.

Further limitations can be found in the selection criteria. For example, focus on “reasons why people are not accessing” sexual health services may have restricted detection of facilitating factors. Facilitators can be deduced from inversing barriers to sexual health services, for example, being able to afford sexual health care, yet independent factors also likely exist. On reflection, facilitators of accessing sexual health care may have deserved more specific attention.

Similarly, restricting to English language papers only could bias our findings. However, despite reported risks of bias when excluding non-English papers in systematic reviews [33], this strategy is not uncommon [34]. Moreover, Morrison et al. [35] found evidence to dispute the existence of bias when implementing language restrictions, whilst Nnate et al. [36] argue the repercussions of language restrictions depend on the review subject. Thus, the negative implications of excluding articles in a language other than English can be questioned. Further, this was necessary from a practical standpoint as funding was not available to support the translation of articles.

Finally, grey literature, such as public reports from charities, were not searched due to time restraints yet may have improved the comprehensiveness of this review [37]. Adams et al. [38] advise the inclusion of grey literature specifically when discussing issues around public health, and as access to sexual health services is argued as a public health issue, not including grey literature may have led to the loss of important perspectives. Consequently, this would be encouraged if this review was replicated.

## Implications and future directions

This review has highlighted the limited availability of good quality, in-depth, firsthand accounts from people with SMI on their experiences accessing sexual health services. Future research should expand on what has been done by conducting open-ended qualitative studies in the wider population of people with SMI, allowing service users to express their experiences and concerns in their own words. This work should take into account diversity in people with SMI, as there are very different attitudes to sex and sexual health between groups, with some groups more at risk of sexual ill-health [39], and certain groups excluded from conversations about sexual health (e.g. older people [40]). Additionally, research should focus on facilitators to accessing sexual healthcare in this group, as well as barriers. Only once we have examined this can we start to find potential solutions to improving access to care.

## Conclusions

This systematic review aimed to identify barriers and facilitators to accessing sexual health services from the perspective of people with SMI. Studies which examined access to HIV and family planning services did so in a way that limited participant responses. While only one study examined barriers and facilitators to accessing generic sexual health services, it did so robustly, though it focussed solely on young women and provided limited data on facilitators. Therefore, future work should focus on examining barriers, and facilitators, to accessing sexual health care in all people with SMI to better identify and address these challenges.

## Supplementary Information

Below is the link to the electronic supplementary material.

Supplementary file1 (DOCX 14 KB)
