# Supplementary material for: Barriers and facilitators to accessing sexual and reproductive health services for people with severe mental illness: a systematic review
Source: Soc Psychiatry Psychiatr Epidemiol. 2025 Feb 28;60(6):1283–95. doi: 10.1007/s00127-025-02844-0 (PMC12162726; doi:10.1007/s00127-025-02844-0)
Supplement: Supplementary file 1 — Supplementary file1 (DOCX 14 KB) [file 127_2025_2844_MOESM1_ESM.docx]

**Online Appendix**

Exact search strategies used in each of the three databases, including their MeSH terms.

APA PsycINFO

1. mental health nurse*.mp.

2. mental health professional*.mp. or exp Mental Health Personnel/

3. psychiatric professional*.mp. or exp Psychiatrists/

4. psychiatric nurse*.mp. or exp Psychiatric Nurses/

5. serious mental*.mp. or exp Serious Mental Illness/

6. severe mental*.mp.

7. schizophreni*.mp. or exp Schizophrenia/

8. schizoaffective*.mp. or exp Schizoaffective Disorder/

9. bipolar*.mp. or exp Bipolar Disorder/ or exp Bipolar I Disorder/ or exp Bipolar II Disorder/

10. manic depress*.mp.

11. mania.mp. or exp Mania/

12. hypomani*.mp.

13. psychosis*.mp. or exp Psychosis/ or exp Affective Psychosis/ or exp Chronic Psychosis/

14. 1 or 2 or 3 or 4 or 5 or 6 or 7 or 8 or 9 or 10 or 11 or 12 or 13

15. sexual health*.mp. or exp Sexual Health/ or exp Sexually Transmitted Diseases/ or exp HIV/

16. sexual health service*.mp.

17. genitourinary medicine*.mp.

18. GUM clinic*.mp.

19. reproductive health*.mp. or exp Reproductive Health/

20. family planning*.mp. or exp Family Planning/

21. contracept*.mp.

22. 15 or 16 or 17 or 18 or 19 or 20 or 21

23. 14 and 22

EMBASE

1. mental health nurse*.mp.

2. mental health professional*.mp. or exp mental health care personnel/

3. psychiatric professional*.mp.

4. psychiatric nurse*.mp. or exp psychiatric nursing/

5. serious mental*.mp.

6. severe mental*.mp.

7. psychosis*.mp. or exp psychosis/ or exp affective psychosis/ or exp schizoaffective psychosis/ or exp depressive psychosis/

8. schizophreni*.mp. or exp schizophrenia/

9. schizoaffective*.mp.

10. bipolar*.mp. or exp bipolar disorder/ or exp bipolar depression/ or exp bipolar I disorder/

11. manic depress*.mp. or exp manic depressive psychosis/

12. mania.mp. or exp mania/ or exp "mixed mania and depression"/ or exp bipolar mania/

13. hypomani*.mp. or exp hypomania/

14. 1 or 2 or 3 or 4 or 5 or 6 or 7 or 8 or 9 or 10 or 11 or 12 or 13

15. sexual health*.mp. or exp sexual health/ or exp Human immunodeficiency virus infection/ or exp Human immunodeficiency virus/ or exp sexually transmitted disease/

16. sexual health service*.mp.

17. genitourinary medicine*.mp.

18. GUM clinic*.mp.

19. reproductive health*.mp. or exp reproductive health/

20. family planning*.mp. or exp family planning/

21. contracept*.mp. or exp contraception/

22. 15 or 16 or 17 or 18 or 19 or 20 or 21

23. 14 and 22

MEDLINE

1. mental health nurse*.mp.

2. mental health professional*.mp.

3. psychiatric professional*.mp.

4. psychiatric nurse*.mp. or exp Psychiatric Nursing/

5. serious mental*.mp.

6. psychosis*.mp. or exp Psychotic Disorders/

7. schizophreni*.mp. or Schizophrenia/ or exp Schizophrenic Psychology/

8. schizoaffective*.mp.

9. bipolar*.mp. or exp Bipolar Disorder/

10. manic depress*.mp.

11. mania.mp. or exp Mania/

12. hypomania.mp.

13. severe mental*.mp.

14. 1 or 2 or 3 or 4 or 5 or 6 or 7 or 8 or 9 or 10 or 11 or 12 or 13

15. sexual health*.mp. or exp Sexual Health/ or exp HIV Infections/ or exp Sexually Transmitted Diseases/

16. sexual health service*.mp.

17. genitourinary medicine*.mp.

18. GUM clinic*.mp.

19. reproductive health*.mp. or exp Reproductive Health/ or exp Reproductive Health Services/

20. family planning*.mp. or exp Family Planning Services/

21. contracept*.mp. or exp Contraception/

22. 15 or 16 or 17 or 18 or 19 or 20 or 21

23. 14 and 22
